# Discrepant Versus Appropriate Responses to Differential Atrial Pacing in Atrioventricular Reentrant Tachycardia: What Is the Mechanism?

**DOI:** 10.1002/joa3.70235

**Published:** 2025-11-30

**Authors:** Can Ozkan, Ozcan Ozeke, Ahmet Korkmaz, Dursun Aras, Serkan Topaloglu

**Affiliations:** ^1^ Department of Cardiology University of Health Sciences, Bursa City Hospital Türkiye; ^2^ Department of Cardiology University of Health Sciences, Ankara Bilkent City Hospital Ankara Turkiye; ^3^ Department of Cardiology İstanbul Medipol University İstanbul Turkey

**Keywords:** atrial overdrive pacing, AVNRT, differential atrial pacing, VA linking

## Abstract

The example illustrates the analysis and measurement of the ventriculo‐atrial (VA) interval following atrial overdrive pacing from both the right atrium and the coronary sinus. At first glance, VA linking does not appear to be present in response to the AOP; however, closer inspection reveals that VA linking is, in fact, observed in the subsequent cycle.

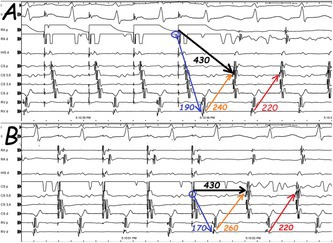

## 1 Ep Rounds

**FIGURE 1:**
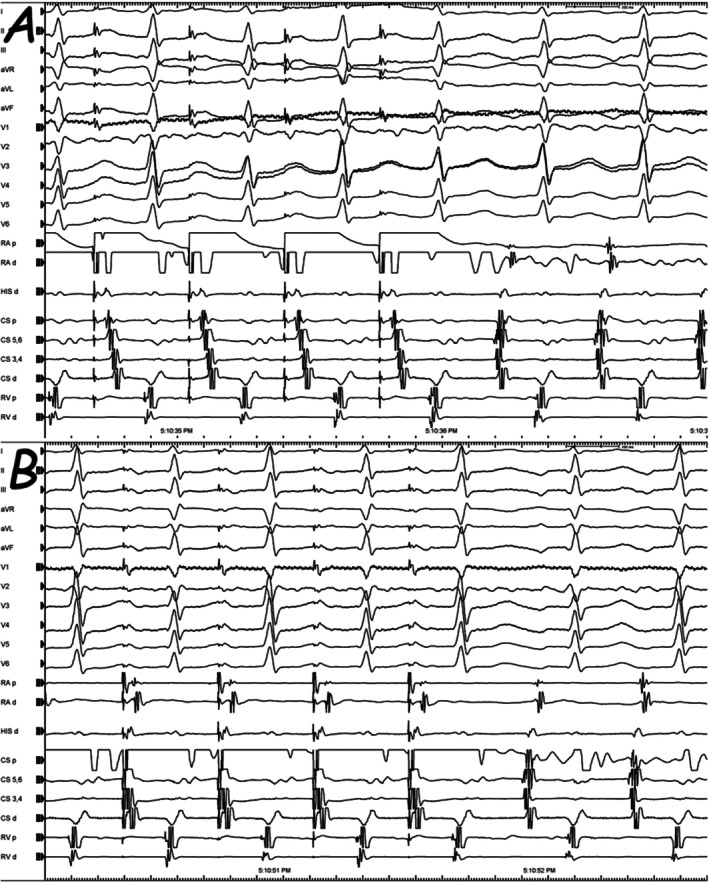
Responses to atrial overdrive pacings from the right atrium (A) and coronary sinus (B) are seen.

A 47‐year‐old woman with documented narrow QRS complex tachycardia (NCT) presented with recurrent palpitations. There was no significant past medical history, and the clinical examination was completely unremarkable. She was brought to the electrophysiology laboratory where catheters were introduced into the right ventricular (RV) apex, His bundle region (His), right atrium (RA), and coronary sinus (CS). Since the pseudo‐VAAV response to ventricular overdrive pacing (VOP) was observed during the electrophysiological study, differential atrial overdrive pacing (AOP) from the RA and CS was applied to confirm the tachycardia mechanism (Figure 1). What is the mechanism of the tachycardia and does this pacing maneuver demonstrate ventriculo‐atrial (VA) linking?

## 2 Discussion

**FIGURE 2:**
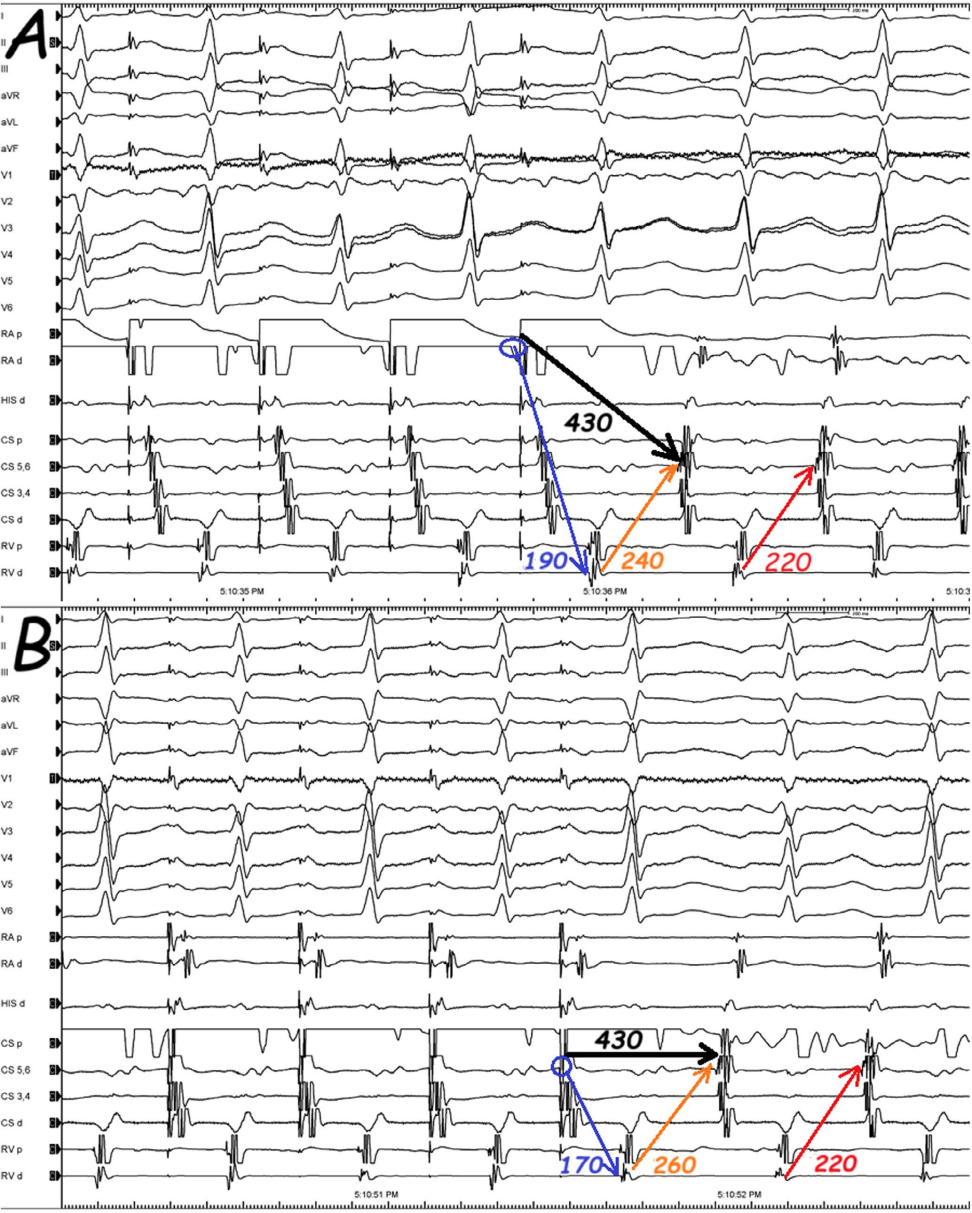
The ventricular entrainment shows a pseudo‐VAAV response.

Despite recent advances in clinical electrophysiology, the diagnosis of atrial tachycardia (AT) originating near Koch's triangle remains challenging. An atrial‐atrial‐ventricular (A‐A‐V) sequence after a VOP is highly specific for AT; however, an A‐A‐V sequence is not rare in atypical AVNRT (Figure 2). A double atrial response is the most common cause of the false positive A‐A‐V response and it was also reported rarely in orthodromic reentrant tachycardia using a decrementally conducting accessory pathway [1]. Therefore, the differential AOP has been used in that situation to differentiate AVNRT from AT. Following the AOP, the VA interval between the last paced conducted ventricular beat and the first spontaneous atrial beat is compared with the VA interval during tachycardia [2]. A fixed VA interval is expected in AVNRT and AVRT, when the first atrial activation following cessation of atrial pacing is linked to the previous ventricular activation (presence of VA linking with difference < 10–20 msn) [2, 3, 4]. The changes in VA interval during AVNRT mostly occur on the first beat after induction of tachycardia [5]. A variable interval would suggest the absence of VA linking and thus the diagnosis of AT because atrial activation is not dependent on ventricular activation [3]. In the conventional method to evaluate VA linking, AOP is performed until NCT is entrained.

**FIGURE 3:**
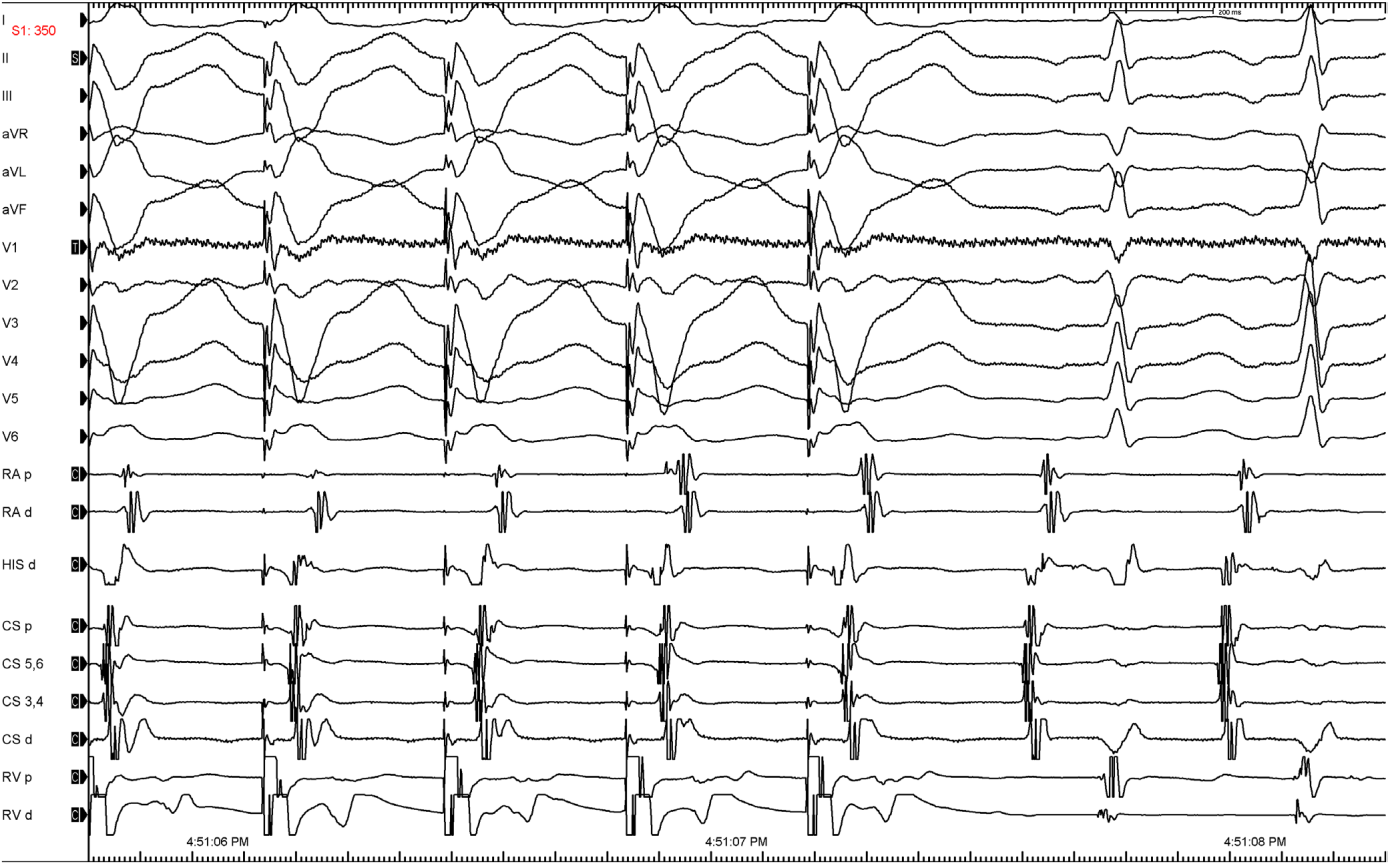
The example illustrates the analysis and measurement of the ventriculo‐atrial (VA) interval following atrial overdrive pacing from both the right atrium (RA) and the coronary sinus (CS). At first glance, VA linking does not appear to be present in response to the AOP (compare the orange arrows in Figure 2); however, closer inspection reveals that VA linking is, in fact, observed in the subsequent cycle (red arrows in Figure 2). Blue arrows indicate the stimulus‐to‐ventricle (SV) interval, black arrows indicate the stimulus‐to‐atrial (SA) interval, and orange arrows indicate the VA interval.

In current tracing, the longer post‐pacing VA interval is probably due to the decremental conduction property in the retrograde slow pathway (orange arrows in Figure 3) [5, 6]. Indeed, it is essential to pace the atrium only slightly faster than the tachycardia rate to prevent far decrementality. However, the VA interval on the second returning tachycardia beat following AOP (red arrows in Figure 3) is only dependent on the retrograde VA conduction and is independent of the site from which the overdrive pacing was achieved [6]. On the other side, it should be kept in mind that VA linking could exist coincidentally in AT and one can observe a fixed VA interval if pacing is performed next to the focus (with a short return cycle); therefore, repeating this maneuver at different AOP cycle lengths and from different atrial sites (to pace remote from the site of earliest activation or to pace from two sites) has been proposed to increase its predictive value. Although the initial impression suggested the absence of VA linking (orange arrows in Figure 3), closer analysis revealed that VA linking was, in fact, present in the subsequent cycle (red arrows in Figure 3). In this individual, the diagnosis was atypical AVNRT, and AV nodal slow pathway ablation was done with no inducible tachycardia thereafter.

## Funding

The authors have nothing to report.

## Ethics Statement

The authors have nothing to report.

## Conflicts of Interest

The authors declare no conflicts of interest.

## Data Availability

The authors have nothing to report.

## References

[1] S. Huseynova , O. Ozeke , I. Erbay , et al., “Four Different Responses to Premature Atrial Conductions During Narrow Complex Tachycardia,” Journal of Cardiovascular Electrophysiology 36, no. 9 (2025): 2427–2430. 40641064 10.1111/jce.70005

[2] M. Maruyama , Y. Kobayashi , Y. Miyauchi , et al., “The VA Relationship After Differential Atrial Overdrive Pacing: A Novel Tool for the Diagnosis of Atrial Tachycardia in the Electrophysiologic Laboratory,” Journal of Cardiovascular Electrophysiology 18, no. 11 (2007): 1127–1133. 17711437 10.1111/j.1540-8167.2007.00928.x

[3] A. H. Kadish and F. Morady , “The Response of Paroxysmal Supraventricular Tachycardia to Overdrive Atrial and Ventricular Pacing: Can It Help Determine the Tachycardia Mechanism?,” Journal of Cardiovascular Electrophysiology 4, no. 3 (1993): 239–252. 8269296 10.1111/j.1540-8167.1993.tb01227.x

[4] R. Selvaraj , K. Nair , A. Subramanian , and K. Nanthakumar , “A Single Atrial Extrastimulus During a Short RP Tachycardia,” Heart Rhythm 7, no. 7 (2010): 997–998. 19717349 10.1016/j.hrthm.2009.06.023

[5] Y. Taniguchi , S. J. Yeh , M. S. Wen , C. C. Wang , F. C. Lin , and D. Wu , “Variation of P‐QRS Relation During Atrioventricular Node Reentry Tachycardia,” Journal of the American College of Cardiology 33, no. 2 (1999): 376–384. 9973017 10.1016/s0735-1097(98)00576-2

[6] A. Sarkozy , S. Richter , G. B. Chierchia , et al., “A Novel Pacing Manoeuvre to Diagnose Atrial Tachycardia,” Europace: European Pacing, Arrhythmias, and Cardiac Electrophysiology: Journal of the Working Groups on Cardiac Pacing, Arrhythmias, and Cardiac Cellular Electrophysiology of the European Society of Cardiology 10, no. 4 (2008): 459–466. 18299309 10.1093/europace/eun032

